# The Genetic Background Effect on Domesticated Species: A Mouse Evolutionary Perspective

**DOI:** 10.1100/tsw.2011.32

**Published:** 2011-02-14

**Authors:** Eli Reuveni

**Affiliations:** Mouse Biology Unit, European Molecular Biology Laboratory, Monterotondo, Italy

**Keywords:** domesticated species, wild species, laboratory mouse strains, evolution, natural selection, population genetics, speciation, genotype-phenotype map

## Abstract

Laboratory mouse strains are known for their large phenotypic diversity and serve as a primary mammalian model in genotype-phenotype association studies. One possible attempt to understand the reason for this diversity could be addressed by careful investigation of the unique evolutionary history of their wild-derived founders and the consequence that it may have on the genetic makeup of the laboratory mouse strains during the history of human fancy breeding. This review will summarize recently published literature that endeavors to unravel the genetic background of laboratory mouse strains, as well as give new insights into novel evolutionary approaches. I will explain basic concepts of molecular evolution and the reason why it is important in order to infer function even among closely related wild and domesticated species. I will also discuss future frontiers in the field and how newly emerging sequencing technologies could help us to better understand the relationship between genotype and phenotype.

## LABORATORY MOUSE STRAINS IN MEDICAL RESEARCH

Although laboratory mouse strains all belong to the same species, *Mus musculus*, hundreds of laboratory mouse strains provide a collection of models owing to their differences in physiological, clinical, morphological, and behavioral traits[1,2,3,4]. Actually, to date, almost all scientific disciplines in medical research use the mouse as a model system to study disease, cancer, immunology, etc. in order to understand mammalian systems in general and as a model for human disease in particular[5,6,7,8]. So why are laboratory mice so particular? Why is it so uncommon to observe the same phenotypic diversity in other mammalian organisms? One of the answers for this question could be addressed by tracing back the evolution of their founders or understanding better the complexity of their chromosomal structure.

## THE GENETIC BACKGROUND OF LABORATORY MOUSE STRAINS

Laboratory mouse strains are believed to have emerged from the human fancy breeding history of three wild-derived subspecies (*M. m. domesticus*, *M. m. musculus*, and *M. m. casteneus*)[9,10,11,12]. While *M. m. musculus* and *M. m. casteneus* subspecies occupy the regions of Eastern Europe and South Asia, the subspecies *M. m. domesticus* inhabits Western Europe and followed human immigration to the New World (America and Australia)[13,14]. Some laboratory strains also include genetic material from *M. m. molossinus,* which is a relatively recent natural hybrid of the *M. m. casteneus* and *M. m. musculus* subspecies[15].

For obvious reasons, the odd breeding history by humans of the classical mice that potentially could be traced back hundreds of years, starting from their early domestication (when humans used mice as companion pets) until the early 20th century (when mice were used in clinical research), was not documented[16]. However, it is accepted that during this breeding history, humans traded mice across continents and probably selected mice that showed particular and desired phenotypic traits[17]. Such long domestication events by humans shaped a unique chromosome structure that could primarily give us some hints for the causality of its vast amount of phenotypes.

We can distinguish between two predominant models that attempt to explain the genetic background of laboratory mouse strains (Fig. 1):

1. The mosaic (polyphyletic)[11,18,19,20] – genome with intervals of low and high variation owing to different ancestral origins (low, intrasubspecific; high, intersubspecific).
2. Intrasubspecific (monophyletic) origin[21] – genome represented mostly by contribution from the same subspecies population origin.

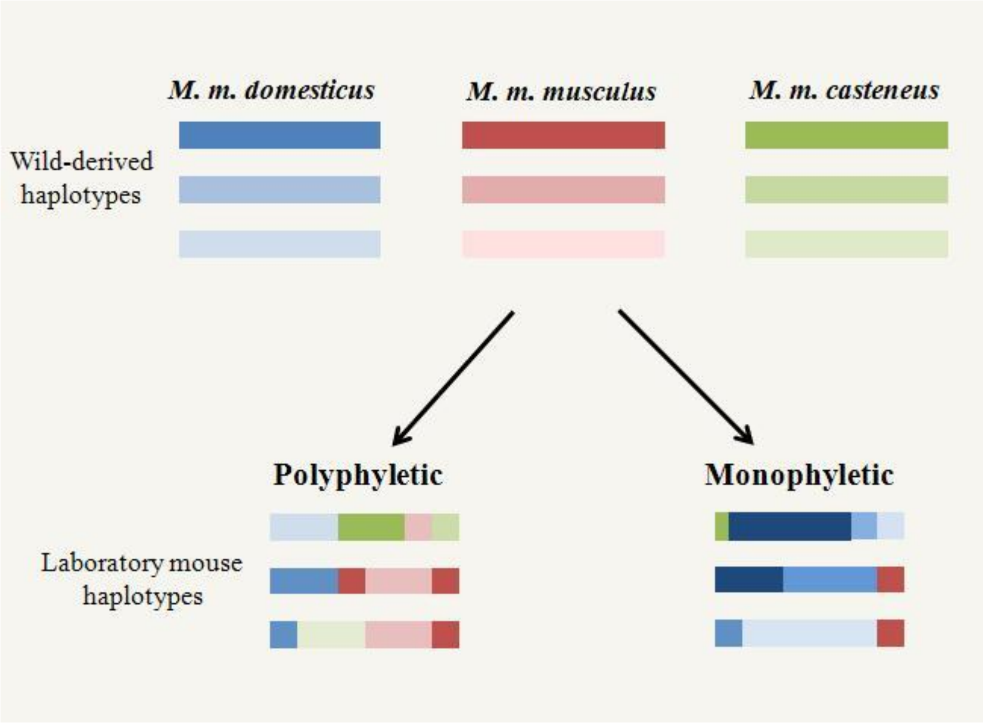

**FIGURE 1.** The figure illustrates two phyletic models describing the genetic background of laboratory mouse strains. Different colors represent distinct subspecies origin, while variants of the same color represent genetic variation within the population origin. While the polyphyletic model suggests greater genetic variability between laboratory strains, the monophyletic model shows that most of the genetic variability between laboratory mouse strains derives from the *M. m. domesticus* subspecies.

The two models are discussed in the following three independent studies. Wade et al.[20] were the first to validate a previous hypothesis of the mosaic model[11] by using random shotgun reads. Their analysis revealed that the vast majority of genetic contribution derives from *M. m. domesticus* and *M. m. musculus*. In another independent study, Frazer et al.[18] used whole genome sequencing analysis of 14 laboratory and four wild-derived strains. This study confirmed the mosaic hypothesis with the following assignment: 68% *domesticus*, 6% *musculus*, and 3% *castaneus*. Interestingly, Frazer et al.[18] estimated that 20% of the single nucleotide polymorphisms (SNPs) present in laboratory mouse strains are invariant in the wild-derived subspecies, leaving the possibility of an additional founder hypothesis. In contrast with the mosaic model, but using the same dataset of Frazer et al.[18], Yang et al.[21] found that 92% of the genome was of *M. m. domesticus* subspecies and concluded that the limited genetic diversity was even more extreme than originally thought, decreasing the research potential of the classical inbred strains. The discrepancy between the two studies could be explained by the fact that both studies made different *a priori* assumptions about the likely number of different founder origins. While Frazer et al.[18] assumed four founders (*M. m. domesticus*, *M. m. musculus*, *M. m. casteneus*, and *M. m. molossinus*), Yang et al.[21] assumed three founders (*M. m. domesticus, M. m. musculus*, and *M. m. casteneus*). The discordance between the last two studies is notable and could be affected by misjudgment of prior assumptions of the hypothetic number of subspecies origin. The fact that 20% of the genome could not be assigned to any of the wild-derived ancestors could be due to the following possibilities: (1) a substantial fraction of the ancestry of classical strains is unsampled, thus the level of genetic diversity within a subspecies population is postulated to be very large; (2) a possible contribution from another *M. musculus* subspecies.

## NATURAL SELECTION AND THE EVOLUTIONARY HISTORY OF WILD MICE

In order to better understand the genetic makeup of the laboratory mouse strains, it is essential to get an insight into the evolutionary history of their founder origin. As previously explained in the text, the *M. musculus* subspecies are known to inhabit naturally across three continents with a stringent barrier to gene flow for the last 1 million years (allopatric species)[22,23,24]. The fact that the branching from their last common ancestor was a relatively long time ago allowed substantial adaptation of mutations to different ecological niches. As a consequence of this, and since the three subspecies are reproductively isolated and they are near to their speciation[12,23,25], natural selection was particularly dominant in each population niche, allowing adaptation of genes and mutations to the surrounding environment. This fact should have a critical effect on the polymorphic spectrum. We would expect to observe strong selective removal of deleterious mutations, i.e., mutations that lower the fitness of an individual carrying the mutation on the one hand and, on the other hand, increase the probability for fixation of advantageous mutations, i.e., mutations that increase the fitness of the organism. In contrast to the polymorphic spectrum between different subspecies, careful examination of the genetic variation between two individuals from the same reproductive population should reveal a more complex organization of mutations in which natural selection is less effective. The last scenario circumvents clear differences between low- and high-fitness mutations, hence their tenability to have effect on the function[26,27]. In general, mutations on the coding regions could be classified into synonymous and nonsynonymous SNPs[28]. While the synonymous mutations maintain the correspondent protein sequence identically, the nonsynonymous mutations can provoke a change in the primary structure of the correspondent protein and, therefore, they are more exposed to removal[29]. Standard procedures in molecular evolution help to classify the strength of natural selection on the coding regions using the $d_N/d_S$ ratio; $d_N$ being the nonsynonymous substitution rates and $d_S$ the synonymous substitution rates[28,30]. In general, natural selection promotes removal of nonsynonymous mutations. Therefore, between species with equal population size and during a long evolutionary period, the $d_N/d_S$ ratio decreases in constant rates and should give some hints for the time elapsed since the divergence of the two branching species (Fig. 2)[29,31]. We can therefore classify two different $d_N/d_S$ curves between laboratory strains: (1) $d_N/d_S$ from within populations (i.e., among *M. m.*

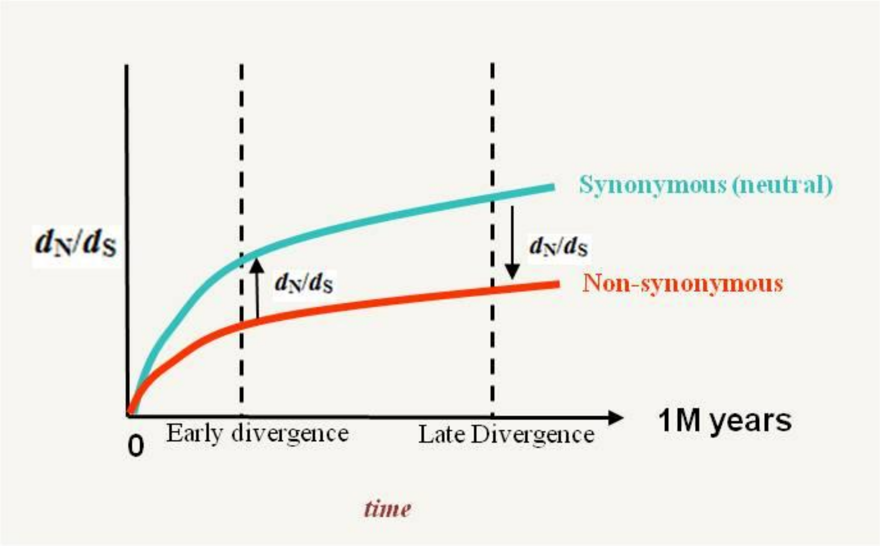

**FIGURE 2.** The figure illustrates the adaptation process of coding mutations as a function of time. In the long run, natural selection should be effective enough to remove deleterious mutations (nonsynonymous, early divergence), while in the short run, the time is not sufficient for substantial fixation (late divergence). As a result, the distribution of the $d_N/d_S$ ratio between mice from the same founder population should be elevated compared to the $d_N/d_S$ distribution between two different subspecies.

*domesticus*) and (2) $d_N/d_S$ between diverged populations (i.e., between *M. m. domesticus* and *M. m. musculus* origin). It is noted in the literature that $d_N/d_S$ can be properly applied to test positive selection (where $d_N/d_S > 1$) only between diverged species and that from within populations, the $d_N/d_S$ ratio could be inflated by segregated nonsynonymous mutations so the $d_N/d_S > 1$ is violated[32,33,34]. Although the interpretation of the "from within population" $d_N/d_S$ may be unjustified to infer positive selection, it still allows us to distinguish between early to late divergence of genes by using a molecular signature of whole-genome $d_N/d_S$[27,31,35]. In addition, it can provide evidence for genes under relaxed selective constraints when the examined species have the same population size, for example *M. musculus* subspecies[36].

## NOVEL METHOD TO UNRAVEL THE GENESIS OF LABORATORY MOUSE STRAINS

Building on the same dataset of Frazer et al.[18], Reuveni et al.[37] developed a novel unbiased method to estimate the number of subspecies founders, excluding prior assumption of ancestral origin. In addition, a pool of genes that may have been under relaxed selective constraints in the ancestral population was proposed. The basic assumption in this study was that a comparison of coding regions between laboratory strains is sufficient to understand the phyletic origin without any clear statement of the correct assignments to the subspecies origin. The approach described in the paper made two prior hypotheses regarding the expected polymorphic spectrum of the laboratory mouse: (1) multifounder origin should be represented by a bimodal distribution when comparing the genetic distance of neutral coding mutations and (2) the $d_N/d_S$ distribution between haplotypes of laboratory strains should keep the molecular signature of its founder origin. The strength behind this approach is that it reduces the likelihood of making a faulty assignment of haplotypes due to evolutionary or sampling effects. Additionally, but more importantly, it allows the drawing of some conclusions regarding the degree of fitness of the genome (or the proportion of mutations that survived selective removal), therefore, to get a more reliable prediction of candidate beneficial mutations. The methods described by Frazer et al.[18] and Yang et al.[21] assigned laboratory mouse haplotypes to their wild-derived ancestors using the latter as the frame sequence. In many cases, the sequenced haplotypes of the wild-derived strains contain large genetic variability that cannot be observed in the laboratory haplotype. For example, individuals from the same population may differ extremely between loci due to the consequence of positive and purifying selection or due to a large genetic drift. Such evolutionary effects can result in one of the following possibilities: (1) the laboratory haplotype is not appropriately classified or (2) the laboratory haplotype is classified as "of an unknown genetic background". Thus, each one of the two possibilities will fail to state the correct ancestral origin.

There are few reasons to believe that the evolutionary approach is less vulnerable to sampling errors. Reuveni et al.[37] demonstrated that pair-wise comparison of the $d_N/d_S$ distribution is similar between the three subspecies for the same gene set and validates the expectation that in a genome-wide manner, natural selection will have, on average, the same impact among each one of the subspecies. However when two rodents from the same subspecies population are examined, a significant up-shift in the $d_N/d_S$ distribution was observed, suggesting that the efficacy of natural selection is proportional to the time since the branching event. Since natural selection may have a variable effect on different genes, gene-by-gene comparison could lead to a faulty statement for the nature of each one of the haplotype origins. However, careful examination of the cumulative $d_N/d_S$ distribution in a genome-wide manner may eliminate errors that could occur due to a single gene affair and support evidence of a common mutability space that is shared between different subspecies. The first interesting finding, and in concordance with their expectations, was that the comparison of 2,000 coding genes of laboratory mouse strains revealed that the $d_N/d_S$ distribution was extremely similar to the one that was observed between different subspecies, but different from the within rodent comparison. This was the first confirmation to the assumption that laboratory mouse strains have inherited genetic materials from several mouse subspecies.

In additional analysis, but this time using neutral (synonymous) polymorphisms to estimate the amount of genetic distance between genes, Reuveni et al.[37] demonstrated that it is possible to assign a cutoff value that distinguishes laboratory mouse haplotypes into close and distant divergence, the closely diverged haplotypes assigned as from an intrasubspecific origin and the distantly diverged haplotypes as from an intersubspecific origin. Interestingly, using different approaches, previous studies have reported the same cutoff value, supporting the validity of the new study[18,20,38]. In contrast to the laboratory mouse haplotypes, the genetic distance of wild-derived strains (within and between population origins) demonstrated Gaussian distribution, indicating a more homogeneous origin. By using clustering approaches, Reuveni et al. confirmed the mosaic hypothesis with the following assignment: 27% same subspecies, 42% two subspecies, and 23% three subspecies, while 8% of the haplotypes could be assigned to a more-than-three subspecies origin. The fact that the evolutionary hypothesis was further validated by the clustering approach provides a novel insight into the genetic makeup of those animals. Furthermore, as a generic approach, this method could be implemented to unravel the genetic makeup of other domesticated species.

## DOMESTICATED SPECIES AND NEW FRONTIERS IN SEQUENCING TECHNIQUES

The cross-continental immigration history of humans was followed by the domestication of a variety of species, including plants, yeast, and animals that were bred in order to improve agricultural crops or as companion pets[39,40,41,42]. In many cases, such domestication forced gene flow between species that were reproductively isolated for thousands of years, creating hybrids that would never have been made under natural circumstances. Usually, these hybrids followed human artificial selection[41] and contain an abnormal spectrum of phenotypes, such as improved yields in crops[43] or showing a large variability in morphological traits. One of the most challenging questions is to understand how such human artificial selection helped to shape those traits, and their association with the genetic variation and allelic interaction. Due to their unusual genetic makeup and as a result of their unusual breeding, domesticated strains provide us with a unique resource to address these questions.

In addition to their ability to exhibit unusual phenotypes, domesticated species can help us to understand the mechanism of reproductive isolation and speciation[44,45,46]. It is already known that certain laboratory mouse strains create hybrid male sterility with other *M. musculus* subspecies[47] and recently it has been shown that *Prdm9* is the causative gene for this allelic incompatibility[48]. The most common model of speciation is known as the Dobzhansky-Muller incompatibilities (for review[49,50]), and usually occurs between two or more alleles that create deleterious interactions in the hybrid, while having a benign or advantageous effect within the same population. The same mechanism of reproductive isolation can be observed in various species with strict geographic barriers and, in the last decade, few speciation genes were identified between species or subspecies of *Saccharomyces*[51], *Drosophila*[52], *Oryza sativa* (Asian Rice[53]), and more. Since genetic barriers can be observed not only between natural species, but also between laboratory strains, unraveling the phylogenic origin of those animals is an elementary task when looking for speciation genes.

Up until recent years, old and highly costly sequencing technologies allowed the full genomic profile of only a restricted number of species to be obtained. This allowed us to study the evolution of the genome only between distantly related organisms, leaving our understanding of its mechanism within closely related species very vague. However due to the reduction in the cost on the one hand and better accuracy of the read calls on the other[54], new sequencing technologies allow us to get the precise genomic profile of organisms down to population scales and thus provide a better resolution to genomic variation, including high-confidence SNP calls[55], copy number variation (CNV)[56], zooming into transcriptomics[57,58], epigenomics[59], or even into the spliceosomic machinery[60]. Moreover, it is inevitable that, in the near future, getting the genomic profile of an organism will be a customary procedure in small laboratories, and it will provide a better understanding of short-term evolutionary mechanisms and better accuracy of the phylogenomic origin of wild species in general or of domesticated animals in particular.

## CONCLUSIONS

Unraveling the genetic background of domesticated animals is a great challenge and one step forward in our understanding of the evolution of selected traits. Being the most-studied mammalian species, the laboratory mouse strains provide an undeniable source of phenotypes that may help us to get a better comprehensive view of the link between the genotype and the phenotype. In this review, I have described a novel approach to unravel the genetic architecture of domesticated animals by using mouse evolution as a probe. From an evolutionary perspective, I have explained that natural selection was particularly effective on the removal of deleterious mutations from the ancestral wild-mice populations. The evidence that many of the polymorphisms are fixed in the laboratory mouse strains confirms the polyphyletic origin of those animals and facilitates the ability to do fine mapping of quantitative trait loci (QTL) underlying complex traits[61].

## ACKNOWLEDGMENTS

This paper was considerably improved by comments from my colleague Brendan Doe from the CNR in Rome.

## References

1. Grubb, S.C., Maddatu, T.P., Bult, C.J., and Bogue, M.A. (2009) Mouse phenome database. *Nucleic Acids Res.* **37,** D720–730.
2. Peirce, J.L., Derr, R., Shendure, J., Kolata, T., and Silver, L.M. (1998) A major influence of sex-specific loci on alcohol preference in C57Bl/6 and DBA/2 inbred mice. *Mamm. Genome* **9,** 942–948.
3. Zhang, Y., Proenca, R., Maffei, M., Barone, M., Leopold, L., and Friedman, J.M. (1994) Positional cloning of the mouse obese gene and its human homologue. *Nature* **372,** 425–432.
4. Cryan, J.F. and Holmes, A. (2005) The ascent of mouse: advances in modelling human depression and anxiety. *Nat. Rev. Drug Discov*. **4,** 775–790.
5. Brown, S.D., Hancock, J.M., and Gates, H. (2006) Understanding mammalian genetic systems: the challenge of phenotyping in the mouse. *PLoS Genet*. **2,** e118.
6. Rosenthal, N. and Brown, S. (2007) The mouse ascending: perspectives for human-disease models. *Nat. Cell Biol*. **9,** 993–999.
7. Friedel, R.H., Seisenberger, C., Kaloff, C., and Wurst, W. (2007) EUCOMM--the European conditional mouse mutagenesis program. *Brief. Funct. Genomic. Proteomic*. **6,** 180–185.
8. Collins, F.S., Rossant, J., and Wurst, W. (2007) A mouse for all reasons. *Cell* **128,** 9–13.
9. Guenet, J.L. and Bonhomme, F. (2003) Wild mice: an ever-increasing contribution to a popular mammalian model. *Trends Genet*. **19,** 24–31.
10. Beck, J.A., Lloyd, S., Hafezparast, M., Lennon-Pierce, M., Eppig, J.T., Festing, M.F., and Fisher, E.M. (2000) Genealogies of mouse inbred strains. *Nat. Genet*. **24,** 23–25.
11. Bonhomme, F., Guenet, J.-L, Dod, B., Moriwaki, K., and Bulfield, G. (1987) The polyphyletic origin of laboratory inbred mice and their rate of evolution. *Biol. J. Linnean Soc*. **30,** 51–58.
12. Boursot, P., Auffray, J.C., Brittondavidian, J., and Bonhomme, F. (1993) The evolution of house mice. *Annu. Rev. Ecol. Syst.* **24,** 119–152.
13. Suzuki, H., Shimada, T., Terashima, M., Tsuchiya, K., and Aplin, K. (2004) Temporal, spatial, and ecological modes of evolution of Eurasian Mus based on mitochondrial and nuclear gene sequences. *Mol. Phylogenet. Evol*. **33,** 626–646.
14. Boursot, P., Din, W., Anand, R., Darviche, D., Dod, B., VonDeimling, F., Talwar, G., and Bonhomme, F. (1996) Origin and radiation of the house mouse: mitochondrial DNA phylogeny. *J. Evol. Biol.* **9,** 391–415.
15. Abe, K., Noguchi, H., Tagawa, K., Yuzuriha, M., Toyoda, A., Kojima, T., Ezawa, K., Saitou, N., Hattori, M., Sakaki, Y., Moriwaki, K., and Shiroishi, T. (2004) Contribution of Asian mouse subspecies Mus musculus molossinus to genomic constitution of strain C57BL/6J, as defined by BAC-end sequence-SNP analysis. *Genome Res.* **14,** 2439–2447.
16. Paigen, K. (2003) One hundred years of mouse genetics: an intellectual history. II. The molecular revolution (1981-2002). *Genetics* **163,** 1227–1235.
17. Morse, H.C. (1978) *Origins of Inbred Mice*. Academic Press, New York.
18. Frazer, K.A., Eskin, E., Kang, H.M., Bogue, M.A., Hinds, D.A., Beilharz, E.J., Gupta, R.V., Montgomery, J., Morenzoni, M.M., Nilsen, G.B., Pethiyagoda, C.L., Stuve, L.L., Johnson, F.M., Daly, M.J., Wade, C.M., and Cox, D.R. (2007) A sequence-based variation map of 8.27 million SNPs in inbred mouse strains. *Nature* **448,** 1050–1053.
19. Wade, C.M. and Daly, M.J. (2005) Genetic variation in laboratory mice. *Nat. Genet*. **37,** 1175–1180.
20. Wade, C.M., Kulbokas, E.J., 3rd, Kirby, A.W., Zody, M.C., Mullikin, J.C., Lander, E.S., Lindblad-Toh, K., and Daly, M.J. (2002) The mosaic structure of variation in the laboratory mouse genome. *Nature* **420,** 574–578.
21. Yang, H., Bell, T.A., Churchill, G.A., and Pardo-Manuel de Villena, F. (2007) On the subspecific origin of the laboratory mouse. *Nat. Genet*. **39,** 1100–1107.
22. Payseur, B.A., Krenz, J.G., and Nachman, M.W. (2004) Differential patterns of introgression across the X chromosome in a hybrid zone between two species of house mice. *Evolution* **58,** 2064–2078.
23. Payseur, B.A. and Hoekstra, H.E. (2005) Signatures of reproductive isolation in patterns of single nucleotide diversity across inbred strains of mice. *Genetics* **171,** 1905–1916.
24. Hunt, W.G. and Selander, R.K. (1973) Biochemical genetics of hybridisation in European house mice. *Heredity* **31,** 11–33.
25. Harr, B. (2006) Genomic islands of differentiation between house mouse subspecies. *Genome Res.* **16,** 730–737.
26. Imhof, M. and Schlotterer, C. (2001) Fitness effects of advantageous mutations in evolving Escherichia coli populations. *Proc. Natl. Acad. Sci. U. S. A.* **98,** 1113–1117.
27. Wyckoff, G.J., Malcom, C.M., Vallender, E.J., and Lahn, B.T. (2005) A highly unexpected strong correlation between fixation probability of nonsynonymous mutations and mutation rate. *Trends Genet.* **21,** 381–385.
28. Yang, Z. (2002) Inference of selection from multiple species alignments. *Curr. Opin. Genet. Dev*. **12,** 688–694.
29. Balbi, K.J. and Feil, E.J. (2007) The rise and fall of deleterious mutation. *Res. Microbiol*. **158,** 779–786.
30. Miyata, T. and Yasunaga, T. (1980) Molecular evolution of mRNA: a method for estimating evolutionary rates of synonymous and amino acid substitutions from homologous nucleotide sequences and its applications. *J. Mol. Evol.* **16,** 23–26.
31. Rocha, E.P., Smith, J.M., Hurst, L.D., Holden, M.T., Cooper, J.E., Smith, N.H., and Feil, E.J. (2006) Comparisons of dN/dS are time dependent for closely related bacterial genomes. *J. Theor. Biol.* **239,** 226–235.
32. Goldman, N. and Yang, Z. (1994) A codon-based model of nucleotide substitution for protein-coding DNA sequences. *Mol. Biol. Evol*. **11,** 725–736.

33. Nielsen, R. and Yang, Z. (2003) Estimating the distribution of selection coefficients from phylogenetic data with applications to mitochondrial and viral DNA. *Mol. Biol. Evol.* **20,** 1231–1239.
34. Kryazhimskiy, S. and Plotkin, J.B. (2008) The population genetics of dN/dS. *PLoS Genet*. **4,** e1000304.
35. Vallender, E.J. and Lahn, B.T. (2007) Uncovering the mutation-fixation correlation in short lineages. *BMC Evol. Biol.* **7,** 168.
36. Salcedo, T., Geraldes, A., and Nachman, M.W. (2007) Nucleotide variation in wild and inbred mice. *Genetics* **177,** 2277–2291.
37. Reuveni, E., Birney, E., and Gross, C.T. (2010) The consequence of natural selection on genetic variation in the mouse. *Genomics* **95,** 196–202.
38. Ideraabdullah, F.Y., de la Casa-Esperon, E., Bell, T.A., Detwiler, D.A., Magnuson, T., Sapienza, C., and de Villena, F.P. (2004) Genetic and haplotype diversity among wild-derived mouse inbred strains. *Genome Res.* **14,** 1880–1887.
39. Hancock, J.F. (2005) Contributions of domesticated plant studies to our understanding of plant evolution. *Ann. Bot.* **96,** 953–963.
40. Purugganan, M.D. and Fuller, D.Q. (2009) The nature of selection during plant domestication. *Nature* **457,** 843–848.
41. Trut, L., Oskina, I., and Kharlamova, A. (2009) Animal evolution during domestication: the domesticated fox as a model. *Bioessays* **31,** 349–360.
42. Liti, G., Carter, D.M., Moses, A.M., Warringer, J., Parts, L., James, S.A., Davey, R.P., Roberts, I.N., Burt, A., Koufopanou, V., Tsai, I.J., Bergman, C.M., Bensasson, D., O'Kelly, M.J., van Oudenaarden, A., Barton, D.B., Bailes, E., Nguyen, A.N., Jones, M., Quail, M.A., Goodhead, I., Sims, S., Smith, F., Blomberg, A., Durbin, R., and Louis, E.J. (2009) Population genomics of domestic and wild yeasts. *Nature* **458,** 337–341.
43. Lippman, Z.B. and Zamir, D. (2007) Heterosis: revisiting the magic. *Trends Genet*. **23,** 60–66.
44. Orr, H.A. (1995) The population genetics of speciation: the evolution of hybrid incompatibilities. *Genetics* **139,** 1805–1813.
45. Dobzhansky, T. (1937) *Genetics and the Origin of Species*. Columbia University Press, New York.
46. Presgraves, D.C. (2010) The molecular evolutionary basis of species formation. *Nat. Rev. Genet.* **11,** 175–180.
47. Forejt, J. and Ivanyi, P. (1974) Genetic studies on male sterility of hybrids between laboratory and wild mice (Mus musculus L.). *Genet. Res.* **24,** 189–206.
48. Mihola, O., Trachtulec, Z., Vlcek, C., Schimenti, J.C., and Forejt, J. (2009) A mouse speciation gene encodes a meiotic histone H3 methyltransferase. *Science* **323,** 373–375.
49. Orr, H.A. and Presgraves, D.C. (2000) Speciation by postzygotic isolation: forces, genes and molecules. *Bioessays* **22,** 1085–1094.
50. Mallet, J. (2006) What does Drosophila genetics tell us about speciation? *Trends Ecol. Evol*. **21,** 386–393.
51. Lee, H.Y., Chou, J.Y., Cheong, L., Chang, N.H., Yang, S.Y., and Leu, J.Y. (2008) Incompatibility of nuclear and mitochondrial genomes causes hybrid sterility between two yeast species. *Cell* **135,** 1065–1073.
52. Phadnis, N. and Orr, H.A. (2009) A single gene causes both male sterility and segregation distortion in Drosophila hybrids. *Science* **323,** 376–379.
53. Long, Y., Zhao, L., Niu, B., Su, J., Wu, H., Chen, Y., Zhang, Q., Guo, J., Zhuang, C., Mei, M., Xia, J., Wang, L., and Liu, Y.G. (2008) Hybrid male sterility in rice controlled by interaction between divergent alleles of two adjacent genes. *Proc. Natl. Acad. Sci. U. S. A.* **105**, 18871–18876.
54. Shendure, J. and Ji, H. (2008) Next-generation DNA sequencing. *Nat. Biotechnol.* **26,** 1135–1145.
55. Li, R., Li, Y., Fang, X., Yang, H., Wang, J., and Kristiansen, K. (2009) SNP detection for massively parallel whole-genome resequencing. *Genome Res.* **19,** 1124–1132.
56. Adams, D.J., Dermitzakis, E.T., Cox, T., Smith, J., Davies, R., Banerjee, R., Bonfield, J., Mullikin, J.C., Chung, Y.J., Rogers, J., and Bradley, A. (2005) Complex haplotypes, copy number polymorphisms and coding variation in two recently divergent mouse strains. *Nat. Genet*. **37,** 532–536.
57. Lee, S., Seo, C.H., Lim, B., Yang, J.O., Oh, J., Kim, M., Lee, B., and Kang, C. (2010) Accurate quantification of transcriptome from RNA-Seq data by effective length normalization. *Nucleic Acids Res.* [Epub ahead of print].
58. Wu, Z.J., Meyer, C.A., Choudhury, S., Shipitsin, M., Maruyama, R., Bessarabova, M., Nikolskaya, T., Sukumar, S., Schwartzman, A., Liu, J.S., Polyak, K., and Liu, X.S. (2010) Gene expression profiling of human breast tissue samples using SAGE-Seq. *Genome Res.* **20,** 1730–1739.
59. Laird, P.W. (2010) Principles and challenges of genome-wide DNA methylation analysis. *Nat. Rev. Genet.* **11,** 191–203.
60. Wang, E.T., Sandberg, R., Luo, S., Khrebtukova, I., Zhang, L., Mayr, C., Kingsmore, S.F., Schroth, G.P., and Burge, C.B. (2008) Alternative isoform regulation in human tissue transcriptomes. *Nature* **456,** 470–476.
61. Mott, R. and Flint, J. (2002) Simultaneous detection and fine mapping of quantitative trait loci in mice using heterogeneous stocks. *Genetics* **160,** 1609–1618.
